# Detection of Cu^2+^ Ions with GGH Peptide Realized with Si-Nanoribbon ISFET

**DOI:** 10.3390/s19184022

**Published:** 2019-09-18

**Authors:** Olena Synhaivska, Yves Mermoud, Masoud Baghernejad, Israel Alshanski, Mattan Hurevich, Shlomo Yitzchaik, Mathias Wipf, Michel Calame

**Affiliations:** 1 Transport at Nanoscale Interfaces Laboratory, Empa–Swiss Federal Laboratories for Materials Science and Technology, Ueberlandstrasse 129, CH-8600 Duebendorf, Switzerland; yves.mermoud@empa.ch (Y.M.); baghernejad@mpip-mainz.mpg.de (M.B.); mathias.wipf@empa.ch (M.W.); 2 Department of Physics, University of Basel, Klingelbergstrasse 82, CH-4056 Basel, Switzerland; 3 Institute of Chemistry, The Hebrew University of Jerusalem, Safra Campus, Givat Ram, Jerusalem 91904, Israel; israel.alshanski@mail.huji.ac.il (I.A.); mattan.hurevich@mail.huji.ac.il (M.H.); shlomo.yitzchaik@mail.huji.ac.il (S.Y.)

**Keywords:** potentiometric sensing, copper, GGH peptide, ion-sensitive field effect transistor, silicon nanoribbons

## Abstract

The presence of heavy metal ions such as copper in the human body at certain concentrations and specific conditions can lead to the development of different diseases. The currently available analytical detection methods remain expensive, time-consuming, and often require sample pre-treatment. The development of specific and quantitative, easy-in-operation, and cost-effective devices, capable of monitoring the level of Cu^2+^ ions in environmental and physiological media, is necessary. We use silicon nanoribbon (SiNR) ion-sensitive field effect transistor (ISFET) devices modified with a Gly–Gly–His peptide for the detection of copper ions in a large concentration range. The specific binding of copper ions causes a conformational change of the ligand, and a deprotonation of secondary amine groups. By performing differential measurements, we gain a deeper insight into the details of the ion–ligand interaction. We highlight in particular the importance of considering non-specific interactions to explain the sensors’ response.

## 1. Introduction

Detecting and monitoring the presence of heavy metal ions in various media, such as drinking and ground water, foodstuff, and, ultimately, in bodily fluids, is a matter of public health due to the recognized impact of these ions on the human body [1,2]. In small quantities, certain heavy metals, like copper (1 mg/kg), manganese (0.17 mg/kg), and zinc (28.6–42.8 mg/kg), are important for our health [3]. Above certain concentrations, heavy metals are however harmful due to their interaction with peptides [2]. This can lead to a modification of the molecular structure of proteins and therefore cause toxicological and carcinogenic effects, affecting the central nervous system, kidneys, and liver, skin, bones, and teeth [4]. The World Health Organization (WHO) and Environmental Protection Agency (EPA) developed guidelines specifying the acceptable amounts of heavy metals in drinking water [5,6].

Here, we focus on the detection of copper, which is an essential element in the human organism. Copper deficiency may cause anemia, neutropenia, and bone abnormalities [7,8]. In addition, elevated concentrations may lead to copper poisoning and damage the liver, brain, and other organs. Abnormal copper concentrations are partially caused by environmental factors, which are linked with the consumption of copper from drinking water [9]. Accurate and reliable detection methods are therefore required to precisely monitor the concentration of copper ions.

Various peptides have been tested for the specific detection of Cu^2+^ ions [10,11,12,13,14]. Using peptides for ion sensing allows the mimicking of biological systems and expands the understanding of important processes, like copper transport realized with the copper-binding site of albumin [11,15]. Aside from established analytical methods to determine Cu^2+^ ion concentrations, such as flame or electrothermal atomic absorption spectrophotometry [16], and spectroscopy [17,18], alternative approaches for specific Cu^2+^ binding using peptides have been tested, such as classical electrochemical methods [19,20,21,22], microcantilevers deflection [23], surface plasmon resonance biosensors [24], and ion-sensitive field effect transistors (ISFET) [25]. A potentiometric detection with ISFETs provides the interesting opportunity to improve our understanding of peptide–ion chelation mechanisms by monitoring charges at functional surfaces bearing self-assembled monolayers (SAM) of receptors. Besides their sensitivity to various targets, achieved with receptor molecules, ISFETs can be fabricated at large in a CMOS compatible process, with low production cost perspectives. However, this approach remains barely explored, with, to the authors’ best knowledge, only a single study attempting to use potentiometric sensing to detect Cu^2+^ ions with peptides [26]. We provide here a systematic characterization of the interaction of copper ions over an extended concentration range, using silicon nanoribbon (SiNR)-based ISFETs, functionalized with glycine–glycine–histidine (Gly–Gly–His, GGH).

**Figure 1:**
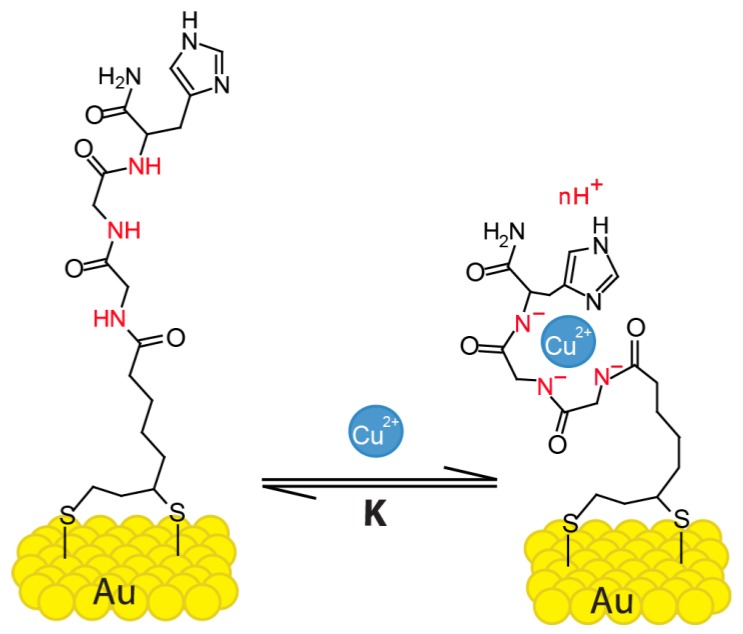
Glycine–glycine–histidine (Gly–Gly–His, GGH) monolayer on a gold surface and the complexation of Cu^2+^ ions. Secondary amines, carrying different charges depending on the electrolyte’s pH, are indicated in red.

The silicon nanoribbons (SiNR) used in this work are separated from the electrolyte solution by an insulating layer of Al_2_O_3_ oxide, and are additionally covered with gold. As shown in our previous studies, this opens the possibility to functionalize the surface with receptors for the detection of various analytes [27,28,29,30]. We investigate here how the net charge at the sensor surface is influenced by the interaction of GGH and Cu^2+^ ions in different buffer solutions over a large analyte concentration range. The peptide Gly–Gly–His (Figure 1) is known to bind copper ions with high affinity. A large range of association constants (10^7^–10^16^) can be found in literature [15,31,32,33,34].The selectivity of the GGH peptide towards Cu^2+^ has been shown in literature [35,36].

Such a high affinity of the peptide to copper ions allows an analyte detection in the low concentration range, from femto- to micromolar. GGH can have different deprotonation states, depending on the pH of the media, which will therefore influence the binding affinity as previously reported, for instance, for peptides with histidine residue [37].

## 2. Materials and Methods

### 2.1. Device Fabrication

The ISFET devices were fabricated from a p-doped silicon-on-insulator (SOI) wafer (Soitec, France) with a buried oxide 145 nm thick, by a top–down approach. The structure of the device is shown in Figure S1. The fabrication process is described in detail in our previous work [27,28,29,30]. Briefly, the top Si(100) layer of the SOI wafer (p-type, 85 nm thick, resistivity 8.5–11.5 Ω) was thermally oxidized in order to grow a 15 nm thick SiO_2_ layer. A device pattern with 48 SiNRs was defined by electron beam lithography (EBL) and etched using a combination of reactive ion etching (SiO_2_ layer) and wet etching (Si device layer) with a mixture of tetramethylammonium hydroxide (TMAH) and isopropyl alcohol (IPA) 9:1 at 45°. The etched NRs were typically 10 µm long, 80 nm high, and 1–25 µm wide. The source and drain of each transistor were doped with boron, and then thermally annealed in a forming gas to activate the dopants (6 min, 950°). As an insulation layer, a 20 nm thick Al_2_O_3_ layer was deposited using atomic layer deposition (ALD) at 225°. Contact pads were opened by wet etching with buffered hydrofluoric acid (BHF), and 300 nm thick Al–Si (1%) pads were deposited by electron beam evaporation and annealed at 450°. To extend the range of application and enable their functionalization with various receptors, SiNRs were covered with 20 nm thick gold film (with a 5 nm chromium adhesion layer) by electron beam evaporation. This coating partially suppresses the response to H^+^ and permits the use of thiol chemistry for surface functionalization [27,28,30]. For additional protection and to create microfluidic channels, the devices were covered with a 2 µm thick layer of SU-8. Finally, the wafer was diced and devices were wire-bonded to the chip carriers. The bonds were sealed with epoxy (Epotek 353ND).

### 2.2. Microfluidic Channels

Microfluidic channels were fabricated in order to reduce the required amount of analyte solutions and to automatize solutions exchange. Polydimethylsiloxane (PDMS) was poured onto a 100 µm thick SU-8 layer on Si, acting as a master, and patterned by EBL with the desired channel structure (two channels in this case). The PDMS was cured for 2 h at 60° and peeled off from the wafer. Two holes at the beginning and at the end of each microchannel were punched in order to connect polytetrafluoroethylene (PTEE) tubes for solution exchange. The whole system was then placed in a larger mold, and more PDMS was added to stabilize the microfluidic chamber.

### 2.3. Surface Functionalization

The devices were cleaned with UV–ozone, rinsed with Milli-Q water, and covered with PDMS microchannels. The devices’ surfaces were functionalized by flushing a LpaGGH solution (83 µM) through one of the microfluidic channels for 12 hours in total, in sequences alternating between 10 s of flushing (solution refresh) and 1000 s of functionalization time [28]. For the time series measurements, a self-assembly process was performed. The ligand solution was applied to the device surface for typically 12 hours. This method has the advantage of sparing material, as only about 50 µl of the ligand solution is necessary for a proper functionalization.

### 2.4. Peptide, Electrolyte, and Cu^2+^ Ion Solutions

For the SAM formation of the gold-coated SiNRs, we used GGH peptides conjugated with a lipoic acid prior to grafting it onto the surface. The GGH peptide functionalized with a lipoic acid was synthesized by a standard HATU-based solid phase peptide synthesis protocol, and its assembly on the surface was characterized with X-ray photoelectron spectroscopy (XPS) as described in previous work [19]. The ammonium acetate solution (5 M), pH buffer Titrisol, and copper (II) nitrate trihydrate were purchased from Sigma Aldrich and diluted with Milli-Q water to the required concentration. The pH was adjusted using KOH and HCl. As previously shown, K^+^ and Cl^−^ ions do not affect the GGH–Cu^2+^ binding [19].

### 2.5. Measurement Procedure

As previously described, two measurement procedures were used: Steady-state and real time. In the first case, the samples were stabilized for 1 min after solution exchange [28,30]. A potential of 100 mV was applied between source and drain. The source–drain current was measured while sweeping the gate voltage, applied with the reference electrode. From the measured transfer curves, we extracted a threshold voltage (*V_th_*) at a given conductance value of 20 nS. Performing such measurement while increasing Cu(NO_3_)_2_ concentration resulted in a relative shift of the transfer curves. We quantified the change in the surface potential of the devices by extracting *V_th_* and plotting it versus concentration. A linear fit to this data gave us the response of the device in mV/decade. As we operated the p-type semiconductor in the accumulation regime, the change in surface potential was given by: $\Delta \Psi =-\Delta V_{th}$ [38].

We measured the response of gold-coated SiNRs, functionalized with the GGH peptide (active surface). To exclude possible contributions from nonspecific interactions, as well as superimposed signals, we also measured the response of the reference, bare gold as control (passive surface). The differential response, characterizing the response due to the active molecular layer, was obtained by subtracting the control SiNRs signal from the active SiNRs signal.

In real-time measurements, a constant source–drain voltage (100 mV) and constant gate voltage (defined by the linear response regime of the device) were applied, and the source–drain current ($I_{sd}$) was monitored versus time. Changes in surface potential while flushing different solutions coud be quantified by normalizing the source–drain current by the transconductance ($g_{m}$) of each individual NR as follows: ΔΨ=−ΔIsdgm. Possible drifts during the measurement were corrected by subtracting the baseline of the electrolyte solution without added copper ions (first five minutes of the measurement) [27]. The measured curves were shifted to 0 for clarity, as we measured the *change* in the surface potential, not the absolute value. At each “step” in concentration, the surface potential shift value for each Cu(NO_3_)_2_ concentration was extracted by averaging the data points after 1 min stabilization time.

## 3. Results

We performed a potentiometric detection of Cu^2+^ ions using SiNR-based ISFETs functionalized with a GGH. We systematically measured the Cu^2+^ response at electrolyte pH ranging from 4 to 8. We used ammonium acetate (50 mM) as electrolyte solution at pH ranging from 5 to 8 and added Cu(NO_3_)_2_ at a concentration ranging from 0.1 fM to 0.1 µM.

**Figure 2:**
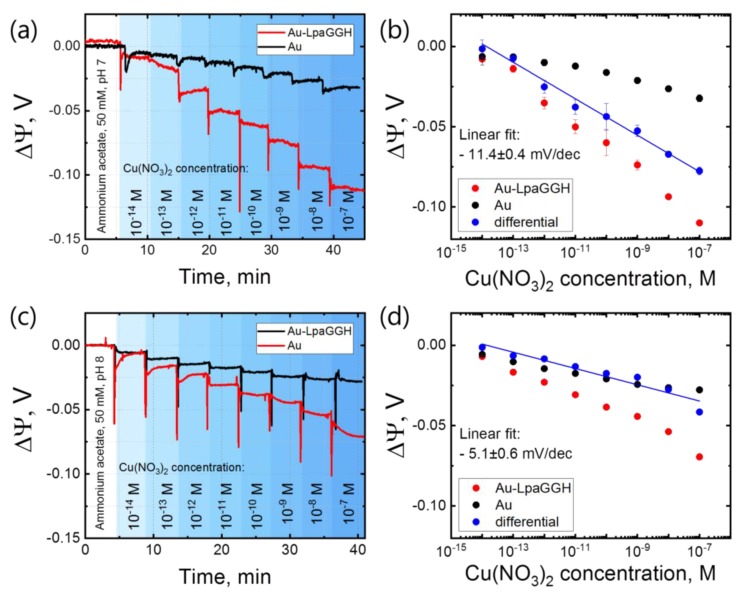
Response of the GGH ligand to Cu(NO_3_)_2_ in the concentration range of 10 fM to 0.1 µM. Real-time measurements of the surface potential shift ΔΨ for increasing concentrations of Cu(NO_3_)_2_ in ammonium acetate (50 mM), pH 7 (**a**) and pH 8 (**c**). For drift correction, a baseline is subtracted from the measured data and the measured curves are shifted to zero (see Methods). (**b**,**d**) Differential response at pH 7 and 8, respectively (see Methods). The reported surface potential values are extracted from “steps” after 1 min settling time, and buffer baseline is shifted to zero.

Figure 2a shows the change in surface potential ΔΨ with increasing Cu(NO_3_)_2_ concentration, starting from ammonium acetate (50 mM, pH 7). The two lines (red and black) represent the Au–GGH (active) and Au (passive) surfaces, respectively. Figure 2b shows the extracted values of the surface potential shift (average value for each concentration after 1 min settling time) versus Cu^2+^ concentration. We observe a negative change in surface potential shift upon binding of Cu^2+^ to the immobilized ligand at pH 7 (about −15 mV/decade). Subtracting the response of the control surface (Au, −4 mV/decade) from the active SiNRs (Au–GGH) results in a differential response of −11 mV/decade (blue dots). We plot the differential response to represent the GGH-Cu system without background signal from Cu^2+^ interacting with the Au surface. At pH 8 we observe a similar trend (Figure 2c,d), with a smaller differential response of −5 mV/decade. The negative differential response appears counterintuitive as the analyte is positively charged. We would indeed *a priori* expect an increase in surface potential with increasing of Cu^2+^ ion concentration, when the surface charge becomes more positive [30]. We however anticipate here that the measured negative change in surface potential reveals a more subtle mechanism upon the complexation of Cu^2+^ ions by the GGH ligand, which leads to a net negative charge of the complex, as sensed by the active surface.

In these experiments, we don’t observe a full Nernstian response. However, we don’t expect that, as the binding reaction is not at the Helmholtz plane, but takes place in the diffuse layer. This limits the response due to screening effects [39], as the charges are further away from the surface, and we measure less change in the potential.

We suggest that the final complex GGH–Cu^2+^ can be negatively charged due to the release of several protons from the ligand upon Cu^2+^ chelation. The GGH peptide has three amide groups that contribute to the complex formation (Figure 1). The nitrogen atom of pyridine in the imidazole ring acts as an anchoring binding site to initiate the Cu^2+^ chelation [10,11,14]. At neutral to basic pH, the Cu^2+^ chelation process sequentially deprotonates the consecutive peptide nitrogen (secondary amides). This process is schematically described in Figure S2.

The Cu^2+^–ligand complex can have four different charge states, depending on the pH, which influences the protonation state of the ligand:

$$L+Cu^{2+}⇄{\left[Cu^{2+}L\right]}^{+2}$$

$$L+Cu^{2+}⇄{\left[Cu^{2+}L^{-1}\right]}^{+1}+H^{+}$$

$$L+Cu^{2+}⇄\left[Cu^{2+}L^{-2}\right]+2H^{+}$$

$$L+Cu^{2+}⇄{\left[Cu^{2+}L^{-3}\right]}^{-1}+3H^{+}$$

At pH 7 and higher, the ligand is neutral. Upon copper binding, the ligand can release up to three protons (secondary amides) and predominantly forms the complex ${\left[Cu^{2+}L^{-3}\right]}^{-1}$ [20,22,23,26]. In addition, Cu^2+^ comes in a variety of ionic forms [40], as the Cu^2+^ hydrolysis depends on pH (occurring at pH 5 and higher). Studies identify five hydrolysis products, with CuOH^+^ and Cu(OH)_2_ (aq.) being prevalent at pH 6 and higher [41]. Cu^2+^ hydrolysis competes with the GGH complexation reaction, affecting the amount of ${\left[Cu^{2+}L^{-3}\right]}^{-1}$ complexes formed and, hence, the surface potential.

Based on our results, we interpret that, upon Cu^2+^ binding to the ligand, secondary amides are deprotonated, and the GGH ligand undergoes a conformational change [19,20,22], bringing an effective negative charge close to the surface (Figure 1). At pH 7 and slightly higher, we expect the formation of −1 charged complexes ${\left[Cu^{2+}L^{-3}\right]}^{-1}$, resulting in a negative surface potential with increasing Cu(NO_3_)_2_ concentration [42]. In slightly basic conditions (ammonium acetate, pH 8), a secondary amine can already be deprotonated. This results in less or even no net gain in charge, and we observe a smaller response. Furthermore, a higher pH leads to more copper (II) hydroxide formation, leading to a lower concentration of free Cu^2+^ ions in the solution, which can also lower the response [43,44].

For the passive surface (Au), the surface potential decreases as well. As emphasized above, several copper hydrolysis products coexist at pH 7 and 8, which shows a non-specific interaction with the Au surface or hydroxyl groups on it.

At pH 6 and lower, we observe no response in the Cu(NO_3_)_2_ concentration range between 0.1 fM and 0.1 µM. The acidic environment prevents the deprotonation of the secondary amine groups of the ligand, lowering the probability for chelation of a cation, thereby leading to a lower affinity for Cu^2+^. At pH 5 and 6, the active Au–GGH devices in the low concentration range show a negligible response (Figure S3).

**Figure 3:**
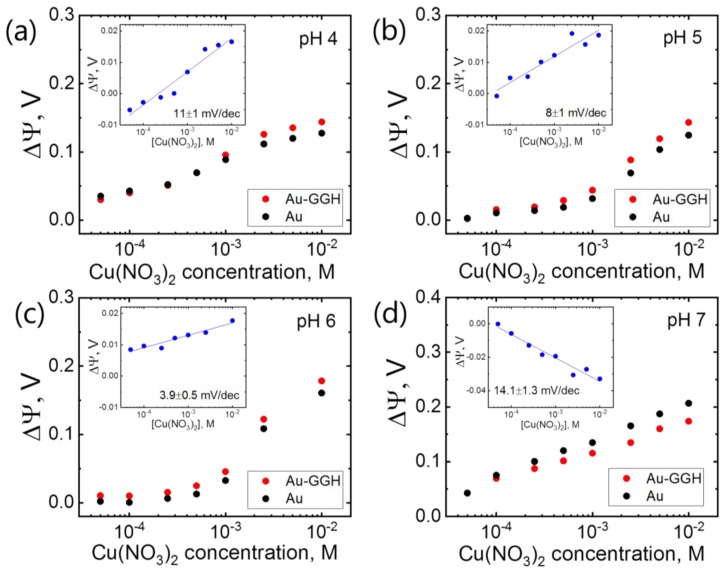
Response to Cu^2+^ ions at high concentrations (0.05–10 mM) in Titrisol buffer at (**a**) pH 4 (105 mM, HCl 0.044, NaOH 0.11, C_6_H_8_O_7_ 0.056 mol/l), (**b**) pH 5 (148 mM, NaOH 0.2, C_6_H_8_O_7_ 0.096 mol/l), (**c**) pH 6 (83 mM, NaOH 0.16, C_6_H_8_O_7_ 0.06 mol/l), (**d**) pH 7, ammonium acetate (50 mM). The signal is averaged over all measured nanoribbons (12 active and 12 control). All insets show the differential response.

In certain conditions, a positive response to Cu^2+^ can be observed. Instead of the expected signal saturation at high Cu(NO_3_)_2_ (from 0.05 mM to 10 mM) concentrations, we observe an increase in the surface potential on all surfaces (Au and Au–GGH), even at low pH. Figure 3 shows the response to Cu(NO_3_)_2_ at pH 4, 5, 6 (Titrisol buffer). The inset shows the differential response of +11 mV/decade. Figure 3b,c shows the response at pH 5 and 6, respectively, +8 mV/decade and +3.9 mV/decade. The positive response indicates that, in this case, another process is prevalent.

We explain this by non-specific adsorption of Cu^2+^ ion to gold at high Cu(NO_3_)_2_ concentrations, leading to a superimposed signal, which is higher than that from the specific interaction process. Furthermore, at pH 6 and lower, the histidine can be protonated, contributing to a more positive net charge of Au–GGH, as compared to the bare Au surface. This is independent, whether chelation is happening or not, as the effective charge of the GGH–Cu^2+^ complex is either neutral or positively charged (+1 or +2) at low pH. This process leads to a clear increase in the surface potential for Au and Au–GGH and, hence, to a positive differential response.

Note that in some cases, a positive response for both the active and the passive wires can lead to an overall negative differential response. This might be interpreted as the expected response from the specific interaction between the analyte and the ligand. We however show in Figure 3d that this situation can also arise when the response is dominated by non-specific interactions. We observe this behavior at high Cu(NO)_2_ concentrations (0.05–10 mM) in ammonium acetate, pH 7, where the differential response is indeed negative, −14 mV/decade. In this case, both surfaces, Au (passive) and Au–GGH (active), show, however, a positive change in surface potential, which indicates that non-specific interactions of Cu^2+^ with the surface dominate the signal from the ligand binding reaction. This again shows the importance of carefully considering all possible contributions to the measured signal.

## 4. Conclusions

We have demonstrated that ISFETs can achieve a quantitative detection of Cu^2+^ ions. Peptides provide a flexible ligand system to ensure a specific detection of the metallic ions. The selected GGH peptide is of particular interest, as it can serve as a model system for the copper-binding site of human serum albumin, which is a major path of copper transport in blood.

We rationalized the GGH peptide–copper ion complexation process by performing differential measurements with SiNR ISFET-based devices and screened different binding conditions, from pH 4 to 8 over an extended concentration range (10^−14^ to 10^−2^ M). We show in particular that the range of reliable operation of ISFETs functionalized with these peptides is restricted to a relatively narrow pH region around neutral pH. The ion–ligand complexation process is indeed affected by pH due to the protonation and deprotonation of secondary amines in the peptide, which can lead to apparently unexpected surface potential changes. We propose a simple mechanism to explain this behavior. We also demonstrate the impact of non-specific adsorption, which dominates the devices’ response in the higher concentration range. This effect still remains too often overlooked, although it substantially affects the overall sensor response. A differential measurement platform, as proposed here, allows this issue to be addressed.

## Supplementary Materials

The following Figures are available online at https://www.mdpi.com/1424-8220/19/18/4022/s1, Figure S1: ISFET Device Structure, Figure S2: Chelation Reactions, Figure S3: Control Measurements at Low Concentration and Low pH, Figure S4: pH Dependence of Copper Ionic Forms.
