# Supplementary material for: Detection of Cu2+ Ions with GGH Peptide Realized with Si-Nanoribbon ISFET
Source: Sensors (Basel). 2019 Sep 18;19(18):4022. doi: 10.3390/s19184022 (PMC6766833; doi:10.3390/s19184022)
Supplement: Supplementary file 1 [file sensors-19-04022-s001.pdf]

Supplementary information

Detection of Cu^{2+} Ions with GGH Peptide Realized with Si-Nanoribbon ISFET

Olena Synhaivska [1,2,*], Yves Mermoud [1,2], Masoud Baghernejad [1,+], Israel Alshanski [3], Mattan Hurevich [3], Shlomo Yitzchaik [3], Mathias Wipf [1] and Michel Calame [1,2,*]

[1] Transport at Nanoscale Interfaces Laboratory, Empa – Swiss Federal Laboratories for Materials Science and Technology, Ueberlandstrasse 129, CH-8600 Duebendorf, Switzerland; yves.mermoud@empa.ch (Y.M.); baghernejad@mpip-mainz.mpg.de (M.B.); mathias.wipf@empa.ch (M.W.)

[2] Department of Physics, University of Basel, Klingelbergstrasse 82, CH-4056 Basel, Switzerland

[3] Institute of Chemistry, The Hebrew University of Jerusalem, Safra Campus, Givat Ram, Jerusalem 91904, Israel; israel.alshanski@mail.huji.ac.il (I.A.); mattan.hurevich@mail.huji.ac.il (M.H.); shlomo.yitzchaik@mail.huji.ac.il (S.Y.)

[+] Current affiliation: Max Plank Institute for Polymer Research, Ackermannweg 10, D-55128 Mainz, Germany

[*] Correspondence: olena.synhaivska@empa.ch (O.S.); michel.calame@empa.ch (M.C.); Tel.: +41-58-765-4076 (O.S.); +41-58-765-4260 (M.C.)

1. ISFET Device Structure

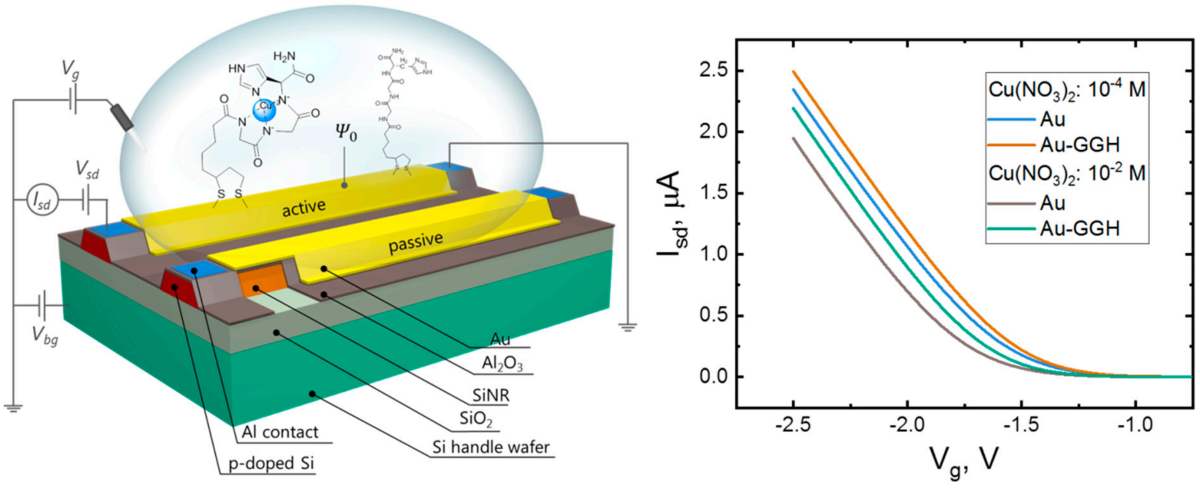

(a) (b)

Figure S1. (**a**) Schematics of the device structure and the measurement setup. The potential of 100 mV is applied between source and drain of each ISFET. The back gate is at 0 V. The gate potential is applied with the reference electrode, and the source-drain current is measured for active (Au-GGH) and passive (Au) surfaces; (**b**) Transfer characteristics of the ISFET for two concentrations of $Cu(NO_3)_2$ in ammonium acetate (50 mM).

2. Chelation Reactions

The reactions, which lead to chelation of Cu^{2+} ion and GGH peptide, are illustrated on the Figure S1.

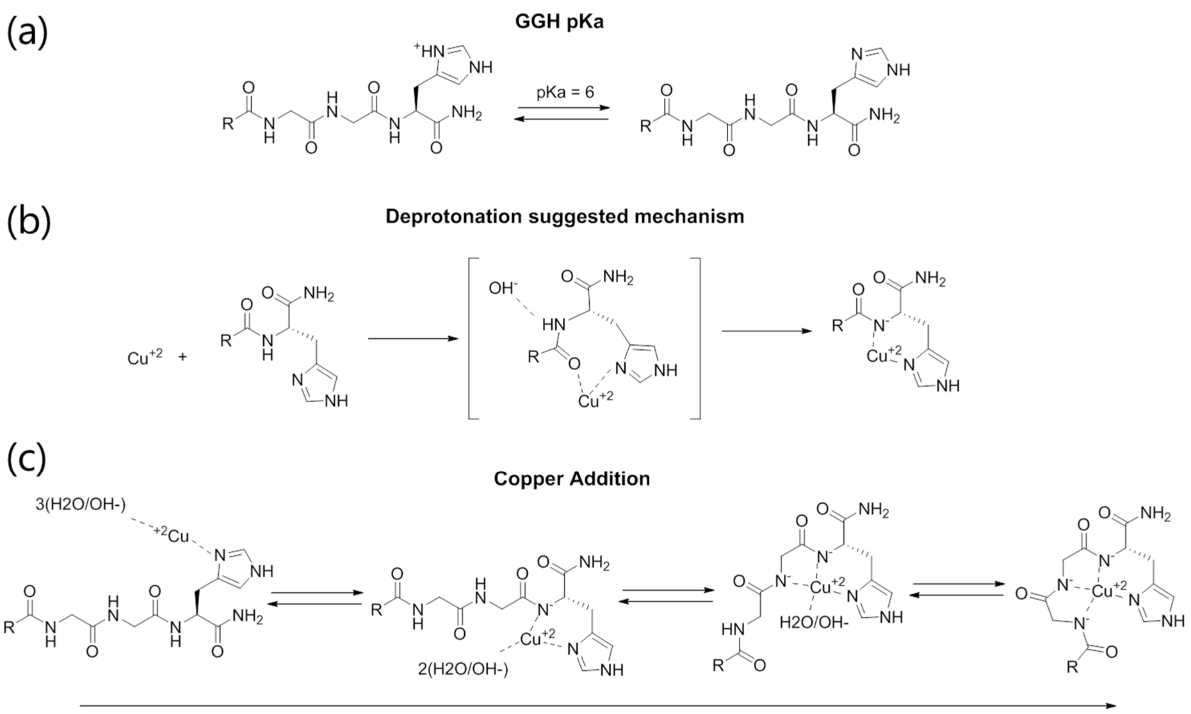

Figure S2. GGH-Cu chelation mechanism: (a) Protonation of imidazole nitrogen, (b) initiation of chelation, (c) chelation process of GGH peptide and Cu^{2+} ion.

3. Control Measurements at Low Concentration and Low pH

In the low concentration range (10 fM – 0.1 μM) in ammonium acetate at pH 5 and 6 (Figure S2) we observe no significant response of the ISFET sensor to Cu^{2+} ions. At these pH values the nitrogen atom of pyridine in the imidazole ring is protonated, which lowers affinity of the GGH peptide to copper ions.

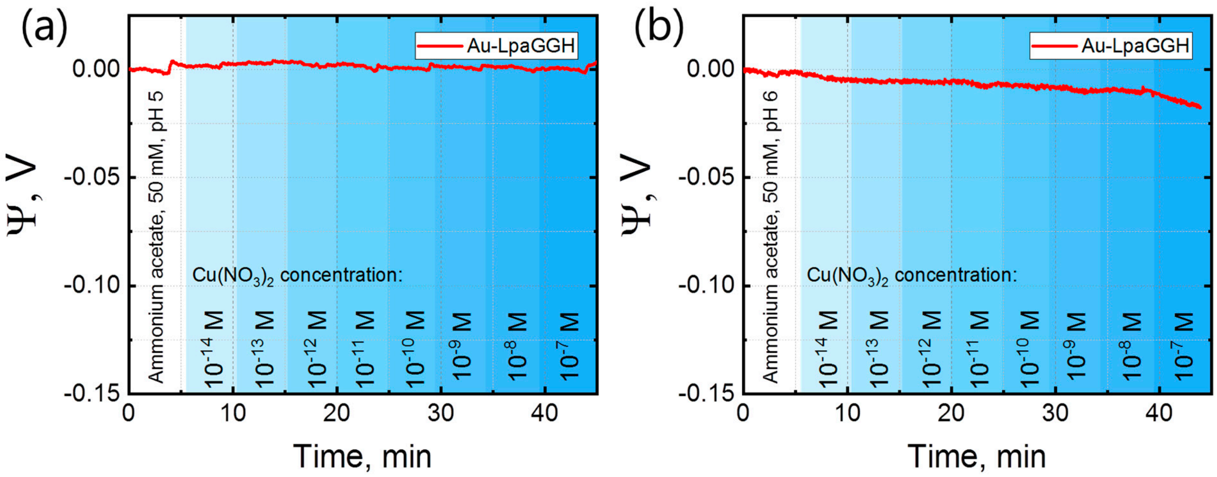

Figure S3. Time-dependent measurements for $Cu(NO_3)_2$ in ammonium acetate (50 mM, pH 5, 6). Concentration range: 10 fM - 0.1 μM.

4. pH Dependence of Copper Ionic Forms

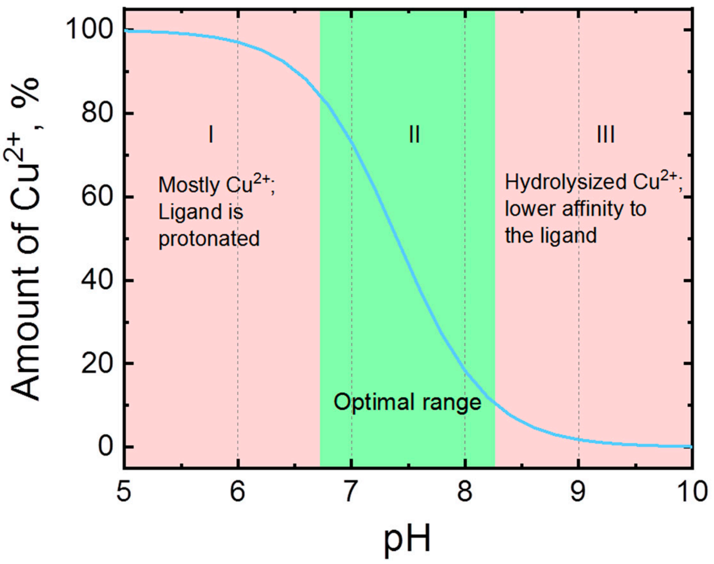

Figure S4. Optimal pH range for Cu^{2+} detection. Qualitative schematics of amount of free Cu^{2+} ions in solution depending on its pH. The plot is divided into three ranges, defining the optimal conditions for Cu^{2+}-GGH interaction.

In the first pH range (I) copper mostly exists in ionic form (Cu^{2+}) [38]. The nitrogen atom in the imidazole ring of the GGH peptide is protonated, which lowers ligand's affinity to copper ions. Therefore not much Cu^{2+} is expected to bind to the ligand in this range. In the second range (II), around neutral pH, nitrogen is deprotonated. Copper in this pH range is not only in ionic state, but also in hydrolyzed form. In this range we obtain a maximal sensor response. In the third pH range of the plot (III), almost no copper is in ionic form. We don't expect chelation in this range, since hydrolyzed copper doesn't bind to ligand with the same affinity as free copper.
